# Hybridization alters growth and migratory life‐history expression of native trout

**DOI:** 10.1111/eva.13163

**Published:** 2020-12-18

**Authors:** Jeffrey T. Strait, Lisa A. Eby, Ryan P. Kovach, Clint C. Muhlfeld, Matthew C. Boyer, Stephen J. Amish, Seth Smith, Winsor H. Lowe, Gordon Luikart

**Affiliations:** ^1^ Wildlife Biology Program, W.A. Franke College of Forestry and Conservation University of Montana Missoula MT USA; ^2^ Flathead Lake Biological Station, Fish and Wildlife Genomics Group Division of Biological Sciences University of Montana Polson MT USA; ^3^ Montana Fish, Wildlife, and Parks University of Montana Fish Conservation Genetics Lab Missoula MT USA; ^4^ U.S. Geological Survey Northern Rocky Mountain Science Center Glacier National Park West Glacier MT USA; ^5^ Montana Department of Fish, Wildlife, and Parks Kalispell MT USA; ^6^ Division of Biological Sciences University of Montana Missoula MT USA

**Keywords:** fitness, growth, hybridization, introgression, invasive species, migratory life history

## Abstract

Human‐mediated hybridization threatens many native species, but the effects of introgressive hybridization on life‐history expression are rarely quantified, especially in vertebrates. We quantified the effects of non‐native rainbow trout admixture on important life‐history traits including growth and partial migration behavior in three populations of westslope cutthroat trout over five years. Rainbow trout admixture was associated with increased summer growth rates in all populations and decreased spring growth rates in two populations with cooler spring temperatures. These results indicate that non‐native admixture may increase growth under warmer conditions, but cutthroat trout have higher growth rates during cooler periods. Non‐native admixture consistently increased expression of migratory behavior, suggesting that there is a genomic basis for life‐history differences between these species. Our results show that effects of interspecific hybridization on fitness traits can be the product of genotype‐by‐environment interactions even when there are minor differences in environmental optima between hybridizing species. These results also indicate that while environmentally mediated traits like growth may play a role in population‐level consequences of admixture, strong genetic influences on migratory life‐history differences between these species likely explains the continued spread of non‐native hybridization at the landscape‐level, despite selection against hybrids at the population‐level.

## 1 INTRODUCTION

Hybridization with introduced species is a serious and growing threat to the conservation of biodiversity and native species worldwide (Allendorf et al., 2001; Grabenstein & Taylor, 2018; Rhymer & Simberloff, 1996). Although natural hybridization can lead to evolutionary novelty and speciation (Grabenstein & Taylor, 2018), human‐mediated introgression can lead to the extinction of native genotypes, loss of locally adapted gene complexes, and outbreeding depression (Araki et al., 2007; Rhymer & Simberloff, 1996; Todesco et al., 2016). Climate change will likely contribute to the continued expansion of human‐mediated hybridization and declines of native taxa (Kelly et al., 2010; Muhlfeld et al., 2014). Therefore, understanding the ecological and evolutionary consequences of introgression is critical for conservation of species threatened with non‐native hybridization. However, there are little data demonstrating the effects of hybridization on ecologically and evolutionarily important traits in the wild, and this lack of scientific data can prevent effective conservation and management (Allendorf et al., 2001, 2004).

Understanding the consequences of hybridization in wild populations is challenging because genetic (G) and environmental (E) factors, as well as their interactions (G × E), can lead to fitness differences among parental and hybrid individuals (Arnold, 1997; Arnold & Martin, 2010; Hunter et al., 2017; Zhang et al., 2019). The effects of human‐mediated hybridization vary depending on the taxa and phenotypic traits examined by researchers (Casas et al., 2012; Fukui, 2020; Muhlfeld, Kalinowski, et al., 2009; Ryan et al., 2009). While laboratory studies can effectively isolate the influence of ancestral genetic differences on phenotypic traits, these studies are often limited to early generation hybrid crosses and lack environmental variation to evaluate E or G × E effects (Drinan et al., 2015; Seiler & Keeley, 2009). Field studies evaluating G × E factors in vertebrates are often limited to association analyses between genotypes and environmental variables (Culumber et al., 2012; Walsh et al., 2016). Of studies that have demonstrated effects of non‐native hybridization on fitness‐related traits in the wild, few have assessed these effects across a range of environmental conditions but see Arnold and Martin, (2010) and Hunter et al., (2017). This limits our understanding of how G, E, and GxE factors affect fitness across heterogeneous landscapes, shaping population‐ and landscape‐level patterns of admixture between native and invasive species (Kovach et al., 2015).

Interspecific hybridization is particularly common in fishes due to limited pre‐ or postzygotic barriers to interbreeding among closely related species, and extensive translocation of non‐native fish species for sportfishing and harvest (Scribner et al., 2001). Rainbow trout (*Oncorhynchus* *mykiss*, RBT) are among the world's most widely introduced fish species (Halverson, 2010) and readily hybridize with native cutthroat trout (*O*. *clarkii* spp.) throughout their native ranges. Hybridization is one of the greatest threats to cutthroat trout subspecies in western North America, including remaining populations of westslope cutthroat trout (*O*. *clarkii* *lewisi*, WCT; Shepard et al., 2005). Hybridization between WCT and non‐native RBT is widespread across a range of environmental conditions, despite strong selection against RBT alleles (Kovach, Hand, et al., 2016; Kovach et al., 2015). Many nonhybridized populations persist in cooler headwater streams (Mckelvey et al., 2016; Muhlfeld et al., 2017), and this genotypic gradient is likely due to both historic stocking locations and environmental variation regulating spread thereafter (Muhlfeld et al., 2017). Similar environmental gradients have been observed in other hybrid zones (Abbott et al., 2018; Walsh et al., 2016), suggesting that environmental variation may partly explain the distribution of admixture across space, but the mechanisms that produce these patterns are rarely understood. Examining the effects of non‐native admixture on individual fitness traits across a range of environmental conditions is needed to gain a more complete understanding of the consequences of hybridization on native biota.

Hybridization between WCT and RBT provides an excellent model to study the effects of hybridization and environmental conditions on fitness outcomes due to the known contributions of both genomic and environmental factors to fitness traits. Growth and migratory life‐history expression affect survival and fecundity in salmonids (Janowicz et al., 2018; Thompson & Beauchamp, 2016). Furthermore, both traits are influenced by genomic (Ali et al., 2020; Hecht et al., 2013; Kelson et al., 2019; Pearse et al., 2019; Prince et al., 2017), environmental (Kanno et al., 2015; Olsson et al., 2006; Thompson & Beauchamp, 2016; Vøllestad & Olsen, 2008), and G × E factors (Bærum et al., 2013; Nater et al., 2018; Yates et al., 2015). Growth is affected by a suite of environmental conditions (Kovach et al., 2016b), but the effects of temperature on growth have been documented in WCT and RBT (Bear et al., 2007). Furthermore, interspecific differences in temperature tolerance suggest that there may be differences in seasonal growth rates between these species that affect the growth of their hybrids in wild populations (Bear et al., 2007; Seiler & Keeley, 2009).

Increased migratory behavior and dispersal of hybrids has been a hypothesized mechanism to explain the rate of spread of RBT admixture (Boyer et al., 2008). Partial migration in salmonids refers to populations where some individuals mature within their natal stream as residents (hereafter, residents) while others migrate to larger water bodies for at least one year before returning to spawn as larger migratory adults (hereafter, migrants). In many taxa, partial migration is thought to be a conditional strategy where genetics, relative body conditions, and environmental context influence the threshold for migration (Berg et al., 2019; Kendall et al., 2015). Since fecundity increases exponentially with length in salmonids (Downs et al., 1997; Janowicz et al., 2018), there are important fitness tradeoffs associated with migratory life‐history behavior.

We examined how individual growth rates and migratory life‐history expression are influenced by proportion RBT admixture (pRBT; G), environmental factors (E), and their interactions (G × E) in three WCT populations inhabiting streams with different thermal and hydrologic conditions. Specifically, we addressed two main questions: (a) Does pRBT affect individual seasonal and annual growth rates and do environmental conditions influence these genetic effects? and (b) Does pRBT affect the propensity to express a migratory life‐history behavior and do environmental conditions influence these genetic effects?

## 2 METHODS

### 2.1 Study sites

**Figure 1:**
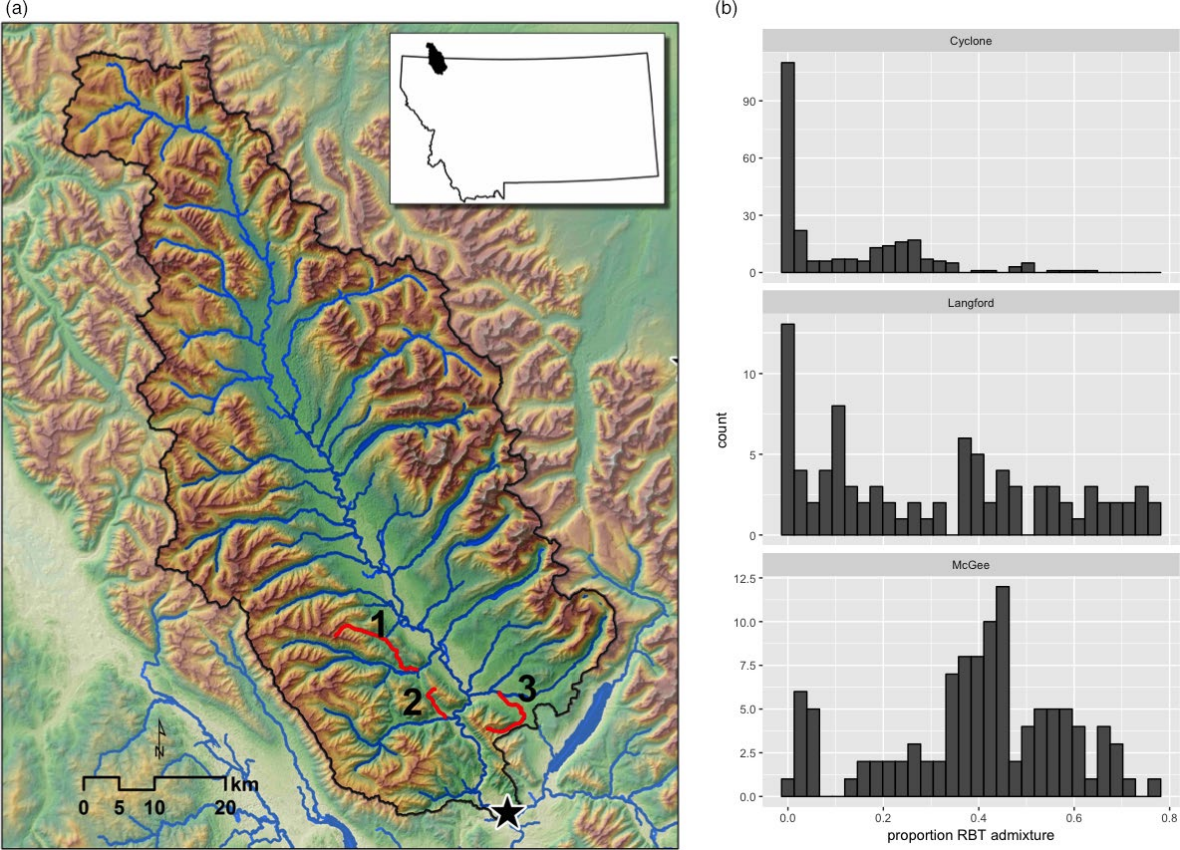
The location of the three focal study sites—Cyclone (1), Langford (2), and McGee (3) Creeks—tributaries to the North Fork Flathead River in Northwestern Montana, USA. (Panel b) shows the distributions of proportion RBT admixture (pRBT) from 2015 sampling efforts. See Figure S4 for distributions of pRBT within each population by sampling year

**Figure 2:**
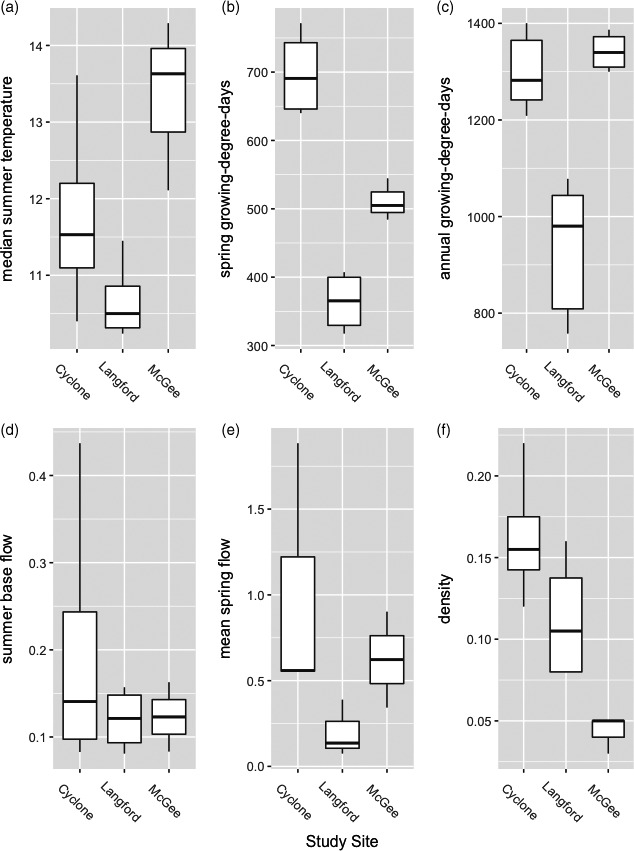
Boxplots of key environmental variables measured in study sites Cyclone, Langford, and McGee Creeks: median summer temperature (°C; a; ANOVA *p* = .031), growing‐degree‐days accumulated during the spring season (b; ANOVA *p* < .00005) and annually (c; ANOVA *p* < .00005), summer base flow (m^3^/s; d; ANOVA *p* = .51), mean spring flow (m^3^/sec; e; ANOVA *p* = .27), and *Oncorhynchus* spp. density (fish/m^2^; f; ANOVA *p* < .0077). See Table S2 for environmental data

We sampled populations of WCT, RBT, and their hybrids in Cyclone, Langford, and McGee creeks in the North Fork Flathead River in northwestern Montana, USA (Figure 1a; 2013–2017). These sites were selected because they contain WCT and hybrids (Figure 1b), and despite being geographically close, differ in key environmental drivers (temperature and flow) thought to influence RBT admixture (Figure 2 and Table S1; Muhlfeld et al., 2009c). Admixture was detected in Langford Creek in the 1980s, but was not detected in Cyclone or McGee Creeks until the 1990s (Muhlfeld et al., 2014). Genetic admixture has been occurring in each population for at least five to ten generations, allowing substantial time for backcrossing and recombination.

### 2.2 Sampling

#### 2.2.1 Question 1: Growth rates

We used mark–recapture to quantify seasonal and annual growth rates across five years (2013–2017). Reaches of each tributary were sampled via electrofishing twice annually (July and October) by completing a single electrofishing pass of each study reach (~2 km). We also conducted two 150‐meter three‐pass depletions in July of each year to estimate trout abundance. For each individual, we measured total length (TL, mm) and mass (g) and collected a tissue sample for genetic analysis. Fish > 70 mm TL were implanted with a unique passive integrated transponder tag (PIT tag) at first capture. We set fixed PIT tag antennae at the downstream end of each study reach and two of the three reaches had PIT tag antennae at the upstream end. Antennae were active from April through November. This allowed us to measure movement out of the study reach and exclude individuals that left the system from our growth analyses. This design allowed us to estimate daily growth rates over three distinct intervals: summer (July*_t_* − October*_t_*), winter/spring (October*_t_* − July*_t_*_+1_; hereafter, spring), and annual (July*_t_* − July*_t_*_+1_ or October*_t_* − October*_t+_*_1_). We obtained growth and genotypic data from 918 individuals for a total of 1,262 growth measurements (Table 1).

**Table 1:** Sample size by year and population for individual growth (2013–2017) and life‐history analyses (2013–2016)

Population	Year	Growth rate analysis				Number adult migrants	Number adult residents	pRBT adult migrants	Life‐history analysis		Number juvenile residents	pRBT juvenile migrants	pRBT juvenile residents
		Summer	Spring	Annual	pRBT				pRBT adult residents	Number juvenile migrants			
Cyclone	2013	24	–	–	0.11	51	44	0.17	0.10	10	129	0.20	0.06
	2014	20	10	15	0.11	39	64	0.18	0.04	4	187	0.07	0.12
	2015	129	33	51	0.12	65	183	0.22	0.08	33	484	0.16	0.11
	2016	105	52	69	0.14	52	157	0.20	0.06	60	546	0.21	0.11
	2017	–	50	106	0.14	–	–	–	–	–	–	–	–
Langford	2013	34	–	–	0.41	26	5	0.37	0.43	42	105	0.40	0.21
	2014	18	15	16	0.32	15	18	0.59	0.38	113	134	0.36	0.26
	2015	43	18	30	0.37	27	40	0.41	0.22	6	131	0.43	0.41
	2016	47	27	27	0.37	61	39	0.47	0.39	31	191	0.45	0.35
	2017	–	15	21	0.36	–	–	–	–	–	–	–	–
McGee	2013	–	–	–	–								
	2014	29	–	–	0.41								
	2015	70	21	27	0.40								
	2016	47	17	18	0.32								
	2017	–	23	32	0.38								

Note

The sample sizes and mean proportion RBT admixture (pRBT) for each year and season of the growth rate dataset are on the left half of the table. The right half shows the sample sizes and mean pRBT for the adult and juvenile datasets in the migratory life‐history analyses.

#### 2.2.2 Question 2: Life‐history strategies

To capture individuals expressing a migratory life‐history behavior, we installed migrant fish traps each spring in Cyclone and Langford Creeks for four years (2013–2016; note: McGee Creek was excluded from this analysis due to insufficient catch of migratory fish in the traps). Migratory adults were captured immigrating into each tributary to spawn and migratory juveniles were captured emigrating downstream leaving the study streams. Traps were checked daily from April through July (primary migratory period) and were removed for the remainder of the year. We followed the same sampling protocol for each trapped individual as for those captured via electrofishing. Individuals that made temporary migrations (e.g., movement across the PIT antennae multiple times within a single season) were removed from the analysis.

### 2.3 Environmental data collection

We collected data on three factors known to affect growth in salmonids: temperature, streamflow, and *Oncorhynchus* spp. density (Figure 2 and Table S1). We measured hourly water temperatures from March through November each year. To characterize the annual and seasonal growing conditions, we calculated median daily temperature and growing‐degree‐days (GDD; summer: suGDD, spring: spGDD, annual: aGDD). We calculated spGDD and aGDD by assuming the growing season begins on the first day of the week in which mean daily water temperature reaches and remains above 5°C and ends on the last day of the week in which mean daily water temperature drops and remains below 4°C (Coleman & Fausch, 2007). Langford Creek was the coldest site and least variable among years (Figure 2a–c). Cyclone and McGee Creeks had warmer and more variable summer temperatures than Langford Creek which had substantial groundwater influence. McGee Creek was the warmest site annually, but Cyclone Creek had much warmer spring conditions than McGee Creek (Figure 2b).

Streamflow was determined with several measures. First, stage height was recorded daily from April through July every year. Second, pressure transducers were added to each site in 2015, recording in‐stream and atmospheric pressure every hour. Finally, stream discharge was measured at regular intervals (at least once a week). Using the stage gage or water pressure measures, we created discharge rating curves to quantify seasonal stream discharge using a polynomial regression. From these data, we computed average summer base flow (~July 1–November 1; m^3^/s), maximum spring flow, and average spring flow (~April 1–July 1). Due to equipment malfunctions, we did not have spring discharge data in 2017, so streamflow was not included in the modeling of spring growth rates. Langford Creek had the lowest spring peak flows, but similar base flows to McGee Creek (Figure 2d,e). Cyclone Creek had the highest mean spring flow; however, McGee Creek had a higher mean peak spring flow.

The abundance of *Oncorhynchus* spp. was estimated using a constant‐effort model (Otis et al., 1978) in the *Fishmethods* package (Nelson, 2016) in Program R. We calculated average wetted stream width at base flow (measured every 50 m longitudinally along the study reaches). The average trout density in each study reach was estimated by dividing the mean abundance estimate by the average wetted width (# fish/m^2^). Densities were highest in Cyclone Creek and lowest in McGee Creek (Figure 2f and Table S1).

### 2.4 Genetic analyses

We genotyped 3,245 individuals across all sites at 650 RBT diagnostic loci that were evenly distributed throughout the rainbow trout genome (see Appendix S1 for detailed laboratory and bioinformatic methods). Proportion RBT admixture (pRBT) was estimated for each individual as the number of RBT alleles/(2 × number of genotyped diagnostic loci). While each population contained nonhybridized WCT, the proportion of the sample that was nonhybridized varied greatly among the study streams (Figure 1b and Table 1). The distribution of pRBT in Cyclone Creek was skewed strongly toward WCT with a median of 0.013 pRBT. Langford and McGee Creek had higher median pRBT (Langford = 0.34; McGee = 0.39) and less skewed distributions of pRBT than Cyclone Creek.

### 2.5 Data analyses

#### 2.5.1 Question 1: Effect of pRBT on growth rate

To test for the effects of pRBT, environmental conditions, and their interactions on seasonal growth rates, we analyzed growth rate in length (mm/day) and mass (g/day) during summer, spring, and annual intervals. We modeled growth separately in each population using multiple linear regression due to differences in the distributions of pRBT, sample sizes, and growth rates among populations. Because these populations are partially migratory, we excluded putative resident adults (>160 mm TL) from the analyses to reduce effects of individuals that have slowed somatic growth (Downs et al., 1997; Janowicz et al., 2018). For each individual, we measured growth based on the observed change in length or mass over the number of days in the sampling interval (spring, summer, or annual).

In addition to pRBT, we considered abiotic and biotic factors known to affect growth rate as potential covariates in our global models. Biotic conditions included individual size at first capture (TL_1_ or W_1_), body condition at first capture (K), and trout density (Table S1). K was estimated as an individual's residual value from a population length‐mass regression (as in Al‐Chokhachy et al., 2019). Abiotic conditions included metrics to characterize seasonal temperature and streamflow (Figure 2 and Table S1).

We hypothesized that stream temperature would have a stronger effect on growth differences between WCT, RBT, and their hybrids (Bear et al., 2007) compared to streamflow or trout density. To avoid including correlated environmental covariates in our growth models, we first identified the optimal temperature metric to include by using the temperature metric whose linear regression model had the lowest AICc (Tables S2,S3). For summer growth the temperature metric with the lowest AICc was median daily temperature, and for spring growth it was spGDD. For annual growth models, we used aGDD as our temperature covariate. We then tested for collinearity between the top temperature metric and streamflow and trout density metrics and only included metrics that were not correlated with temperature (*r* < .6).

We used the *nlme* package in Program R (Pinheiro et al., 2017) to conduct linear modeling following the model selection protocol of (Zuur et al., 2009). We modeled growth (both length and mass) in each population and season separately. We first found the best supported variance structure correcting for pRBT and year since there was evidence of heteroscedasticity in the residuals plotted against pRBT. We then performed top‐down model selection beginning with a saturated global model (all hypothesized covariates and interactions), removing the least significant term until only well‐supported variables remained. In addition, we tested several null models (without pRBT). To avoid selecting models with spurious associations and uninformative parameters (Arnold, 2010), we only considered models that contained all supported parameters (*p* < .05; Tables S4–S12).

#### 2.5.2 Effect of pRBT on migratory life‐history behavior

To test for the effect of pRBT on migratory life‐history behavior, we used a combination of migratory trap and electrofishing data collected from 2013 to 2016. We used migrant fish traps and fixed PIT tag antennae to identify migratory adults (immigrating to spawn) and migratory juveniles (emigrating from study streams) moving between April 1 and July 1 of each year. Summer electrofishing surveys (June 20–July 31) were used to identify resident individuals each year. To assign individuals as adults or juveniles, we used a TL threshold of 160 mm (juveniles ≤ 160 mm; adults > 160 mm; Downs et al., 1997; Janowicz et al., 2018). We captured and genotyped 206 and 129 migratory adults and 107 and 192 migratory juveniles from 2013 to 2016 in Cyclone and Langford creeks, respectively (Table 1). We used generalized linear modeling (GLM) with a logit link function to test for an effect of pRBT on the probability an individual was a migrant (1 = migratory, 0 = resident).

In addition to pRBT, we considered other biotic and abiotic factors thought to influence migratory life‐history behavior in salmonids. We included TL and K in our global model as both have been shown to influence propensity for migration (Ferguson et al., 2019; Kendall et al., 2015). We included capture year in our model as a blocking factor to account for any environmental conditions that might lead to variation migratory behavior over time. We used the *lme4* package in Program R (Bates et al., 2015) to model the probability an individual was a migrant in both the adult and juvenile datasets for each population. We performed the similar model selection process as described in the previous section and compared supported models using AICc (Tables S13,S14).

## 3 RESULTS

### 3.1 Effect of RBT admixture on growth rate

We found evidence that growth rates were influenced by G, E, and GxE interactions. First, seasonal growth rates were influenced by site. Growth was highest during the spring season in all populations, and mean spring growth was similar among populations (Figure S1). Mean summer growth was more variable among sites. Langford Creek had the highest mean summer (1.8× Cyclone & 1.2× McGee) and annual growth rates (1.4× Cyclone & 1.02× McGee). Cyclone Creek had the lowest mean growth rates in all seasons.

Variation in environmental factors influenced seasonal growth rates, and temperature was the most consistent environmental factor generally having a positive effect on growth rates. Variation in temperature significantly influenced growth rates in Cyclone and Langford creeks, but not in McGee Creek (Tables S4–S12). Spring and summer temperature generally had significant positive effects on growth rates (Tables S4, S5, S7, S8). Only McGee Creek had a significant negative effect of fish density on summer growth rates (Table S6b).

**Figure 3:**
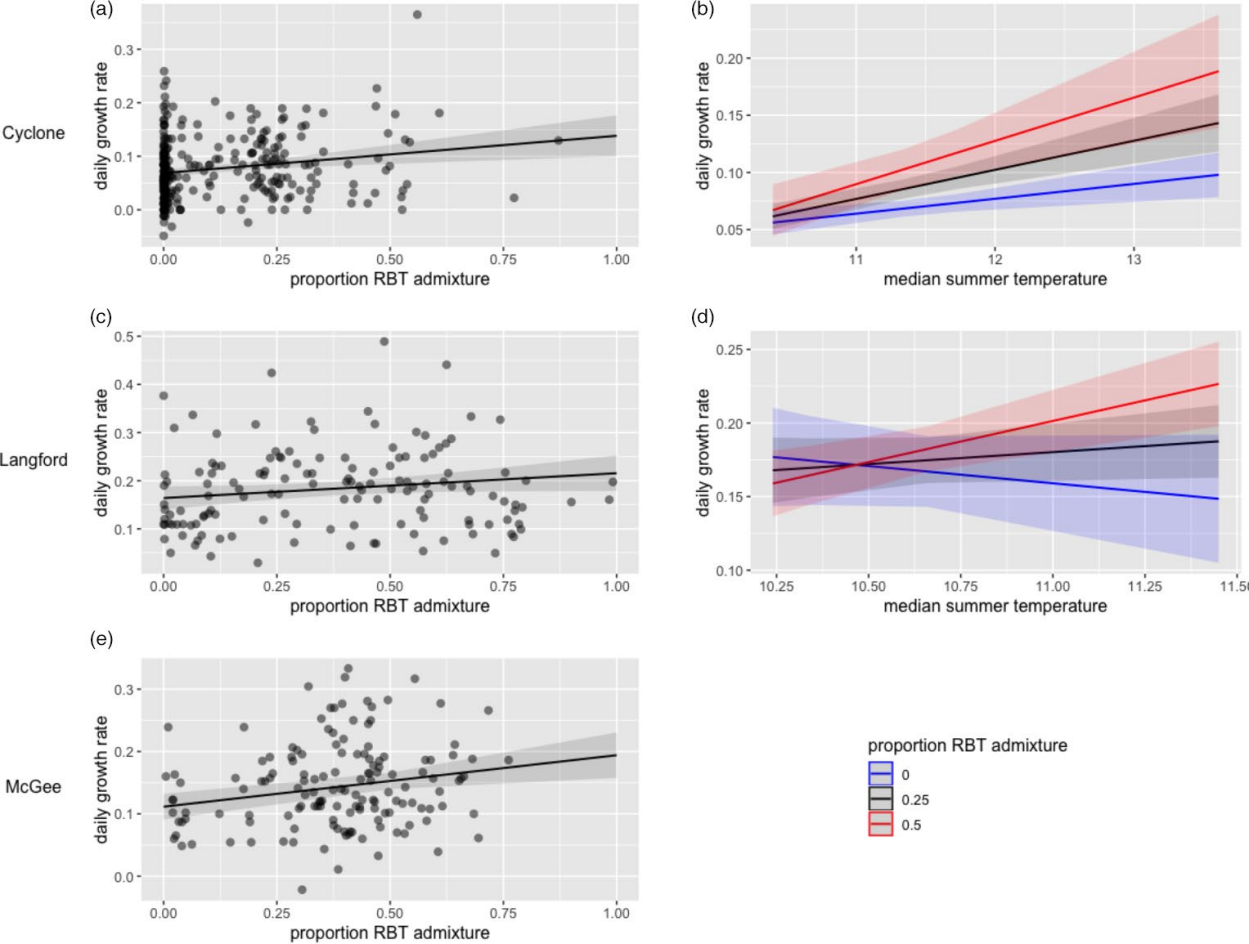
The left column shows the predicted relationship between proportion RBT admixture (pRBT) and daily growth rate from the top supported models of summer growth rate (mm/day) in each population: Cyclone (a), Langford (c), and McGee Creeks (e). The right column shows the interaction between pRBT and median summer temperature on growth rates for Cyclone (b) and Langford Creeks (d), where the different lines depict different pRBT (nonhybridized WCT—blue, 0.25 pRBT—black, and 0.5 pRBT—red)

The effect of pRBT on growth rates (G) generally had a positive effect during warmer conditions and a negative effect during cooler conditions. We focused on the effects of pRBT on growth rates where results for growth in length and weight were consistent; we reported on results for length (mm/day) while results for mass are available in Appendix S2. During the warmer summer season, pRBT was positively associated with growth rates in all populations (Figure 3a,c,e). However, during the cooler spring season, there were significant negative effects of pRBT on spring growth rates in Langford Creek and no effect in Cyclone Creek (Figure S2b and Tables S7–S9). On an annual basis, pRBT had a significant positive effect on growth rates in Cyclone Creek only (Figure S2a and Tables S10–S12). Langford and McGee Creeks showed no evidence of pRBT on annual growth rates.

Both within a season and comparing its effects among seasons, pRBT had a positive effect on growth rates under warmer conditions and negative (or neutral) effects under cooler conditions. Specifically, we found strong evidence that GxE interactions influenced summer growth rates. Higher summer temperatures led to increasingly higher growth rates as individual pRBT increased in Langford and Cyclone Creeks (positive summer temperature × pRBT interaction; Figure 3b,d and Tables S4a,S5a). Furthermore, if we compare the effect of pRBT on growth rates among seasons (summer and spring), pRBT consistently had a positive effect on growth rates during the warmer season (summer) and a negative or neutral effect during the cooler season (spring).

### 3.2 Effect of RBT admixture on migratory life‐history behavior

**Figure 4:**
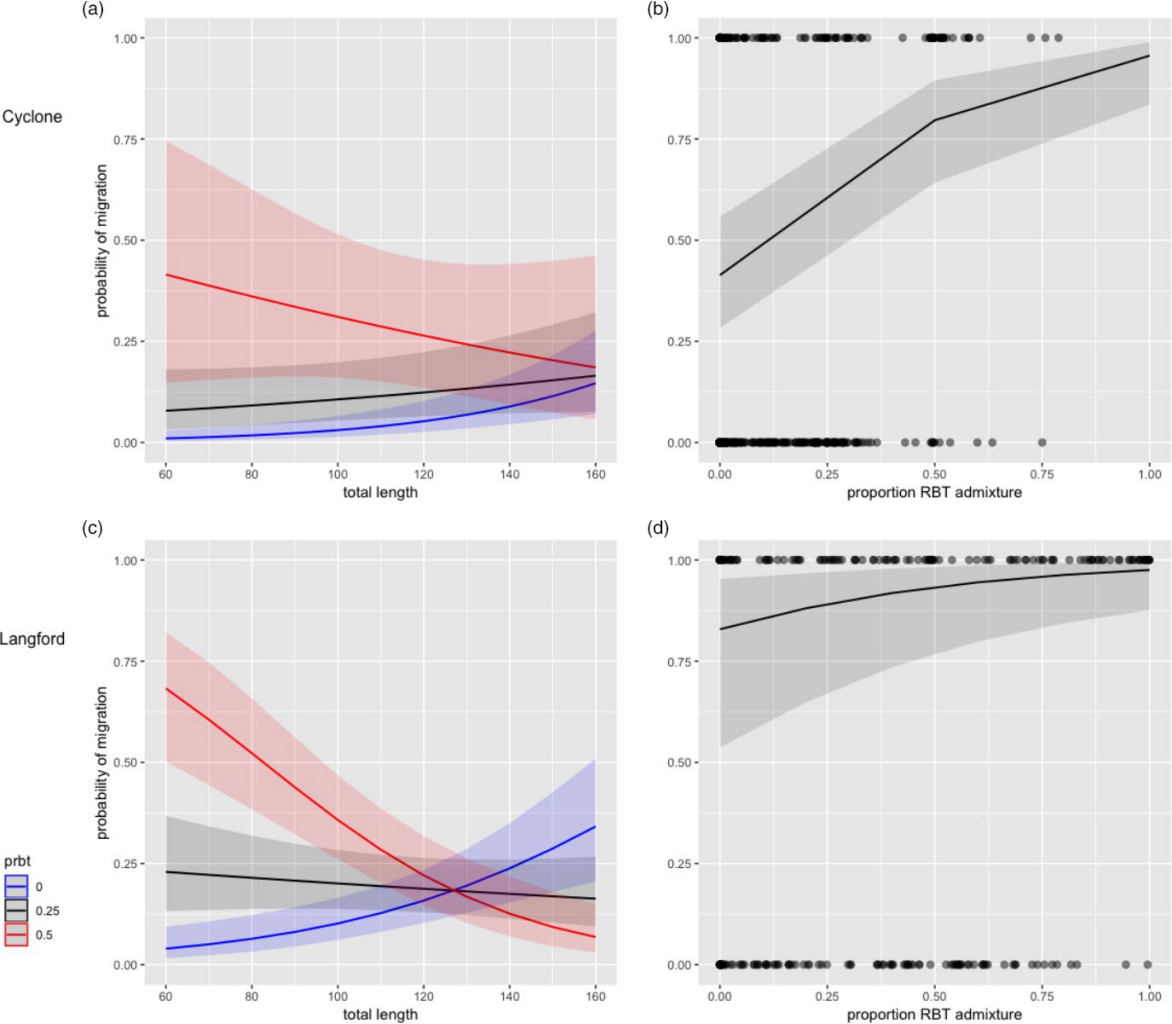
Predicted relationship between proportion RBT admixture (pRBT) and the probability of expressing a migratory life history in Cyclone and Langford Creeks for juveniles (left) and adults sampled (right). Plots (a) and (c) show the negative effect of pRBT on probability of emigration as total length (mm) increases for nonhybridized WCT (blue), 0.25 pRBT individual (black), and 0.5 pRBT individual (red). Plots (b) and (d) show the positive effect of pRBT on the probability of captured adults being migratory

The probability of migration was consistently and positively associated with pRBT for juveniles and adults (Figure 4). In both Cyclone and Langford Creeks, the covariate structure for the juvenile and adult models included pRBT, K, TL, and year effects (Tables S13,S14). Generally, the coefficients had the same direction of effect (positive or negative), although the magnitude of effect sizes differed slightly among populations. For example, adult migration probability increases ~31% and ~20% for individuals with pRBT of 0.4 versus WCT in Cyclone and Langford Creeks, respectively. Juvenile emigration probability was also influenced by a significant interaction between pRBT and TL (Figure 4a,c). More specifically, the probability of emigration increased with TL for WCT and individuals with low pRBT. For moderately or highly admixed hybrids, the probability of emigration decreases as TL increases. This relationship indicates that hybrids are more likely to emigrate at smaller sizes than WCT; however, the strength of this relationship differs between Cyclone and Langford Creeks.

## 4 DISCUSSION

Our study is one of the first to evaluate how human‐mediated hybridization and environment conditions interact (G × E) to affect individual fitness‐related traits in wild vertebrate populations. We found that non‐native admixture influenced both seasonal growth rates and migratory life‐history behavior. Growth rates were influenced by G × E interactions associated with thermal conditions, while the propensity for individuals to migrate was greater in individuals with higher proportion non‐native admixture, independent of environmental conditions. Our analysis of growth rates revealed G × E interactions at different temporal scales; not only did pRBT positively interact with stream temperature to influence summer growth rates, but it had opposing effects on growth across different seasons. These results highlight how environmental variation among populations can influence the outcomes of non‐native hybridization on ecologically and evolutionarily important traits and suggest that non‐native hybridization has the potential to disrupt locally adapted phenotypic optima with links to local fitness outcomes.

Non‐native admixture led to increasingly higher summer growth rates for hybrids across a range of temperatures suitable for WCT growth. Median summer temperature at our study sites ranged from 10.2 to 14.3°C, a range well within the growth limits for WCT and RBT which have similar thermal optima (Bear et al., 2007; WCT = 13.7°C, RBT = 13.2°C). Since pRBT positively interacted with temperature in our models, it is likely that RBT and hybrids experience a summer growth advantage across much of the Flathead River basin. For example, even at the coldest site pRBT positively affect growth (Langford Creek: median summer temperature ≤ 10.7°C). Higher growth rates during summer conditions support the hypothesis that hybrids may have metabolic or physiological advantages (Rasmussen et al., 2012) that may impart a competitive advantage over cutthroat (Seiler & Keeley, 2009).

However, lower summer growth rates may be less consequential to fitness outcomes if WCT make up this lag in growth during the spring. In the populations with cooler spring conditions (Langford and McGee Creeks), pRBT was negatively associated with spring growth rates, and there was no pRBT effect on annual growth. This suggests that a pRBT × season (G × E) interaction could exist in similar streams. Cyclone Creek had no effect of pRBT on spring growth and accumulated ~48% and ~27% more spring growing‐degree‐days than Langford and McGee Creeks, respectively (ANOVA *p* < .00005). If cooler stream temperatures contribute to the negative effect pRBT on spring growth, it is not surprising that this relationship was not significant in Cyclone Creek.

Climate change induced shifts in thermal and hydrologic regimes will likely create more favorable conditions for RBT and hybrids in the Flathead River basin (Jones et al., 2014). Altered thermal and hydrologic conditions over the last three decades have already been associated with increased expansion of hybridization in the Flathead River (Muhlfeld et al., 2014). However, the Flathead River basin (and much of its current range) are predicted to remain within a suitable thermal range for WCT (Isaak et al., 2015), providing quality habitat for WCT in the face of climate change if the threat of hybridization were neutralized. Nevertheless, increasing summer stream temperatures may strengthen the positive effects of pRBT on summer growth rate as RBT have a wider scope‐for‐growth and can continue to grow at temperatures exceeding 20°C (Bear et al., 2007). These climatic shifts could alter the growth patterns seen in other seasons, specifically warmer spring conditions may favor higher growth rates in RBT and hybrids during that season as well.

Differences in seasonal growth patterns due to pRBT may still play an important role in fitness, even when there are no net annual differences in growth. Higher annual growth may impact life‐history traits such as age‐at‐maturation, migration versus residency, and fecundity (Janowicz et al., 2018; Kendall et al., 2015); seasonal growth rates can have important implications on body condition or size‐selective seasonal survival (Al‐Chokhachy et al., 2019; Carlson et al., 2008). Investigating the effects of non‐native admixture on seasonal survival rates is a critical next step in understanding the complex interactions between these fitness traits and the environment.

The consistent effect of pRBT on probability of migration adds crucial knowledge to the literature on life‐history differences among these species (Corsi et al., 2013; Kovach et al., 2015; Muhlfeld et al., 2009b). Our results add further support to the conclusions of Boyer et al. (2008) that the dispersal rate of hybrids is likely higher than WCT. Our results also parallel those found by Kovach et al. (2015) showing juvenile hybrids tend to emigrate from natal streams at a smaller size than WCT. Together, the influence of pRBT on life‐history phenology and migratory behavior suggest a probable mechanism for the continued expansion of RBT introgression in native WCT populations. Given the size differences that typically exist between resident and migratory trout (Downs et al., 1997), increased migratory behavior of hybrids is a life‐history trade‐off with important consequences for reproductive potential and fitness.

Our findings of a genetic driver of migratory life‐history variation align with recent findings from salmonids (Hecht et al., 2013; Kelson et al., 2019; Pearse et al., 2019) and other vertebrate taxa (mammals—Berg et al., 2019; McDevitt et al., 2009; birds—Delmore et al., 2016; Ralston et al., 2019). Interestingly, many studies of partially migratory populations suggest the slower growing individuals are most likely to adopt the migratory strategy because they cannot acquire enough energy locally and must seek more productive areas to grow and mature (Berg et al., 2019; Ferguson et al., 2019; Yates et al., 2015). Our results do not indicate a consistent association between the effects of non‐native admixture on growth rates and its effects on migration. While our study creeks differed in the effect of pRBT on annual growth, pRBT had a consistent effect on the probability of migration across sites. Our study is one of few across vertebrate taxa to demonstrate effects of hybridization on partial migration behavior. Our findings parallel those of Yates et al., (2015) where genetic differences explained more variation in the probability of freshwater maturation in Atlantic salmon (*Salmo* *salar*) than environmental treatments. Determining the true mechanisms underlying migratory life‐history behavior is challenging (Berg et al., 2019; Kendall et al., 2015); perhaps the genetic associations with migration in hybrid zones represent interspecific differences in the switch point between migrant and resident forms (Sloat et al., 2014). New genomic techniques (i.e., admixture mapping, genome‐wide association analyses) should provide greater insight into the genomic structure of partial migration life history and the effects hybridization has on it.

These data provide further evidence that hybrid dispersal is the critical mechanism driving the spread of hybridization between these species, not species‐specific differences in thermal tolerance or growth rates (Kovach et al., 2015, 2017). The complex nature of growth might make it misleading or uninformative when trying to predict fitness outcomes and the spread of non‐native admixture. While juvenile growth is generally considered an important fitness trait linked to competition (Seiler & Keeley, 2009), survival (Carlson et al., 2008), maturation, and life‐history strategy (Kendall et al., 2015), our study shows that the effects of pRBT on growth rates are not likely linked to differences in migratory behavior or population‐level pRBT (selection). For example, Cyclone Creek was the warmest stream and the only population that had a positive effect of pRBT on annual growth rates, yet it had a lower proportion of migrants (~31% migratory adults; Table 1) and the lowest population‐level pRBT (Figure 1b). This suggests factors other than stream temperature and growth rates influence population‐level pRBT. Our study provides important supporting evidence of these hypotheses explaining the patterns of fitness and hybrid zone expansion. Beyond WCT, these results suggest that dispersal of rainbow trout hybrids, which is facilitated by their preponderance for a migratory life‐history, may be a key factor explaining spatial and temporal patterns of hybridization in other cutthroat subspecies.

We found that fitness‐related traits in WCTxRBT hybrid zones are influenced by genetic, environmental, and genotype‐by‐environment interactions. Broadly, it is critical to understand how G, E, and GxE factors affect fitness in human‐mediated hybrid zones and the role of these factors in shaping both population‐level and landscape patterns of admixture between native and invasive species. Studies demonstrating how environment influences the effects of non‐native hybridization on important phenotypic traits in wild populations are rare. This lack of research limits our understanding of drivers of admixture across heterogeneous landscapes and may hinder the ability of resource managers to take appropriate conservation actions. In this system, changes in the seasonal growth patterns and migratory life‐history behavior might represent the disruption of locally adapted phenotypic optima and have unforeseen consequences on evolutionary trajectories and population persistence. Environmentally influenced traits may be important for determining fitness outcomes and population‐level admixture; however, traits under environment‐independent, genetic influence are likely more important drivers for the spread of non‐native hybridization.

## CONFLICT OF INTEREST

We declare we have no competing interests.

## AUTHORS’ CONTRIBUTIONS

JS lead field sampling, laboratory, and statistical analyses. RK contributed to study design, data collection, and data analysis. LE contributed to study design and data analysis. SA and SS contributed to genetic marker development and laboratory analyses. WL and GL contributed funding and study design. MB and CC contributed to study design, funding, and data collection. JS wrote the first draft of the manuscript, and all authors contributed to revisions.

## ETHICAL APPROVAL

All field sampling was done within the guidelines of the Institutional Animal Care and Use Committee permit no. AUP 007‐19.

## Supporting information

Appendix S1‐S2

## Data Availability

The datasets used in all analyses can be found at Dryad: https://doi.org/10.5061/dryad.q2bvq83hg
