# Supplementary material for: Hybridization alters growth and migratory life‐history expression of native trout
Source: Evol Appl. 2020 Dec 18;14(3):821–33. doi: 10.1111/eva.13163 (PMC7980306; doi:10.1111/eva.13163)
Supplement: Supplementary file 1 — Appendix S1‐S2 [file EVA-14-821-s001.docx]

Supporting Information:

Appendix S1 - Methods

1. *Genetic analyses*
2. *Laboratory protocols*

To estimate individual genome wide proportion RBT admixture (pRBT), we extracted DNA using the SPRI bead extraction protocol described in Ali et al. [1]. DNA quality (260/280 ratio) and quantity were measured using a Nanodrop 2000 Spectrophotometer (Thermo Scientific, Waltham Massachusetts; Any use of trade, firm, or product names is for descriptive purposes only and does not imply endorsement by the U.S. Government). The concentration of double stranded DNA was measured using QuantIt Picogreen assays (Thermo Fisher Scientific, Waltham, Massachusetts) after diluting samples to less than 20ng/ul.

Sequencing libraries were prepared using the bestRAD and Rapture (RAD-capture) protocols [1] using 50 nanograms of input DNA for each sample. RAD libraries were sheared to an average fragment size of 350 base pairs using a Covaris E220 Ultrasonicator (Covaris Inc., Woburn, Massachusetts). Libraries were amplified for 12 cycles using a plate specific indexing primer, purified using Ampure XP beads, and quantified using Quantit Picogreen assays. Plates were pooled in groups of 6 (83ng from each library) before enriching for 3015 RAD loci informative for estimating admixture coefficients. Enrichment was performed using a custom Mybaits target enrichment kit (V3., Arbor Biosciences, Ann Arbor, Michigan). This panel included a combination of baits complementary to previously identified RAD loci containing WCT polymorphic SNPs and RBT, WCT, Yellowstone cutthroat trout species diagnostic SNPs [2–5]. Loci were chosen for capture based on their genotyping quality, reliability for distinguishing each (sub)species and even distribution across the assembled rainbow trout genome [6]. Pooled libraries were amplified post-capture for 10-12 cycles and quantified using Quantit Picogreen assays before being sequenced 12 libraries/lane on an Illumina HiSeq X (Novogene Corporation, Sacramento, California).

1. *Bioinformatics and genotype calling*

Read quality was evaluated using FASTQC v0.11.5 and duplicates reads were removed using the clone_filter program from Stacks v1.44 [7]. Sequencing adapter contamination was removed from reads using Trimmomatic and reads were truncated whenever the mean Phred score across a window of 4 nucleotides dropped below q15. We required reads to be greater than 60 bp after applying the trimming steps above. Properly oriented fastq files were demultiplexed by individual barcode using process_radtags v1.44. Reads were mapped to the OmyK_1.0 RBT reference genome [GCF_002163495.1; 6] using bwa-mem and resulting sam files were sorted, converted to bam format, and indexed using samtools v1.4 [8]. We then used HaplotypeCaller v3.7 to generate gVCF files for each individual, combined gVCF files across individuals using CombineGVCFs v3.7, and called genotypes using GenotypeGVCFs v3.7 [9]. The resulting VCF file was filtered using vcftools [LGPLv3; 10].

RBT diagnostic loci were used in the following analyses. Genotypes were set to missing if the genotype quality score was less than 30 and read depth was less than 7. In addition, an allele balance between 0.25 – 0.75 and a minimum read depth of 10 was required for all heterozygote genotypes. After filtering, loci were removed if they were missing genotypes in more than 10% of individuals, and individuals were dropped from the analysis if they did not have genotypes at greater than 20% of remaining loci. Proportion RBT admixture (pRBT) was estimated for each individual as the number of RBT alleles / (2 * number of genotyped diagnostic loci).

We genotyped 3,245 individuals across all sites. We retained 650 RBT diagnostic loci after filtering. The median number of loci per individual was 536, and all individuals were genotyped at a minimum of 160 RBT diagnostic loci. While each population contained non-hybridized WCT, the proportion of the sample that was non-hybridized varied greatly among the study streams (Fig 1b, Table 1). The distribution of pRBT in Cyclone was skewed strongly towards WCT with a median of 0.013 pRBT. Langford and McGee Creeks had higher median pRBT (Langford = 0.34; McGee = 0.39) and less skewed distributions of pRBT than Cyclone Creek.

Supplementary References:

1. Ali OA, O’Rourke SM, Amish SJ, Meek MH, Luikart G, Jeffres C, Miller MR. 2016 Rad capture (Rapture): Flexible and efficient sequence-based genotyping. *Genetics* **202**, 389–400. (doi:10.1534/genetics.115.183665)

2. Amish SJ, Hohenlohe PA, Painter S, Leary RF, Muhlfeld C, Allendorf FW, Luikart G. 2012 RAD sequencing yields a high success rate for westslope cutthroat and rainbow trout species-diagnostic SNP assays. *Mol. Ecol. Resour.* **12**, 653–660. (doi:10.1111/j.1755-0998.2012.03157.x)

3. Hohenlohe PA *et al.* 2013 Genomic patterns of introgression in rainbow and westslope cutthroat trout illuminated by overlapping paired-end RAD sequencing. *Mol. Ecol.* **22**, 3002–3013. (doi:10.1111/mec.12239)

4. Hand BK *et al.* 2015 Genomics and introgression: Discovery and mapping of thousands of species-diagnostic SNPs using RAD sequencing. *Curr. Zool.* **61**, 146–154. (doi:10.1093/czoolo/61.1.146)

5. Kovach RP *et al.* 2016 Vive la résistance: genome-wide selection against introduced alleles in invasive hybrid zones. *Proc. R. Soc. B Biol. Sci.* **283**, 20161380. (doi:10.1098/rspb.2016.1380)

6. Berthelot C *et al.* 2014 The rainbow trout genome provides novel insights into evolution after whole-genome duplication in vertebrates. *Nat. Commun.* **5**. (doi:10.1038/ncomms4657)

7. Catchen JM, Amores A, Hohenlohe P, Cresko W, Postlethwait JH. 2011 *Stacks* : Building and Genotyping Loci *De Novo* From Short-Read Sequences. *G3&amp;#58; Genes|Genomes|Genetics* **1**, 171–182. (doi:10.1534/g3.111.000240)

8. Li H, Handsaker B, Wysoker A, Fennell T, Ruan J, Homer N, Marth G, Abecasis G, Durbin R. 2009 The Sequence Alignment/Map format and SAMtools. *Bioinformatics* **25**, 2078–2079. (doi:10.1093/bioinformatics/btp352)

9. McKenna A *et al.* 2010 The Genome Analysis Toolkit: A MapReduce framework for analyizing next-generation DNA sequencing data. *Genome Res.* , 1297–1303. (doi:10.1101/gr.107524.110.20)

10. Danecek P *et al.* 2011 The variant call format and VCFtools. *Bioinformatics* **27**, 2156–2158. (doi:10.1093/bioinformatics/btr330)

Appendix S2 - Supplemental Figures and Tables


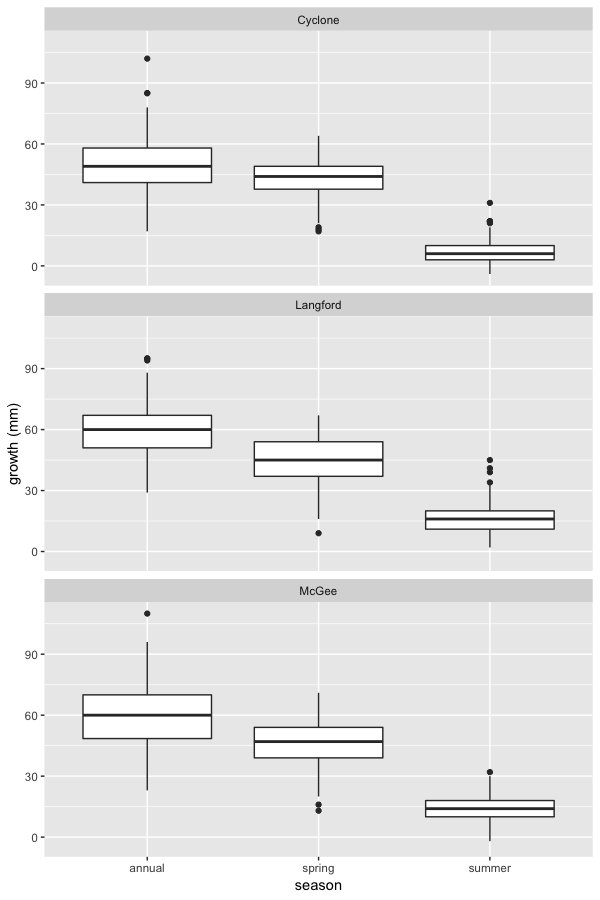


**Fig S1**: Boxplots of annual, spring, and summer growth in length (mm, top) for each population for Cyclone (top), Lanford (middle), and McGee (bottom) Creeks. Growth during the spring season is significantly higher than summer growth in all populations (t-test, *p* < 0.00001).


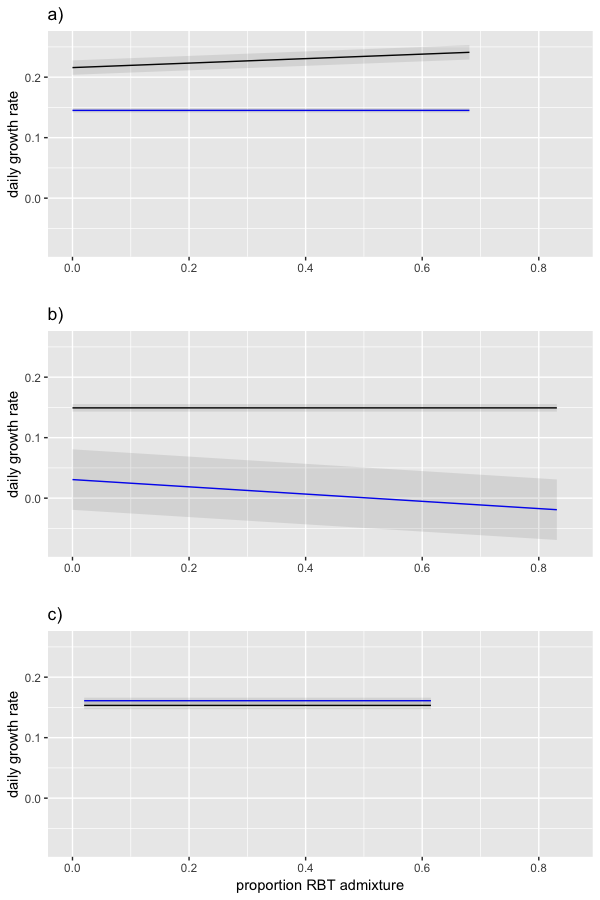


**Fig S2**: Predicted relationship between proportion rainbow trout (RBT) admixture (pRBT) and daily growth rate (mm/d) for a) Cyclone, b) Langford, and c) McGee Creeks. The blue line shows the predicted effect of pRBT on spring daily growth rates (mm/d) and the black line represents the predicted effect of pRBT on annual daily growth rates (mm/d; see Fig S7 for full data).


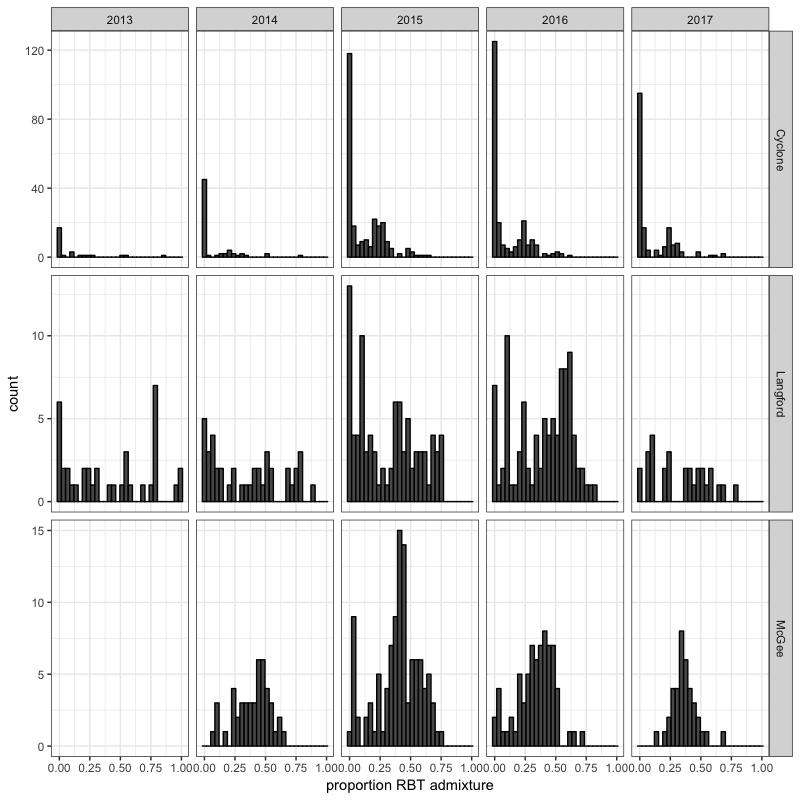


**Fig S3:** Distributions of proportion rainbow trout (RBT) admixture in each population across the study period (2013-2017) for Cyclone, Langford, and McGee Creeks. Distributions within each population remain relatively stable across the study period.


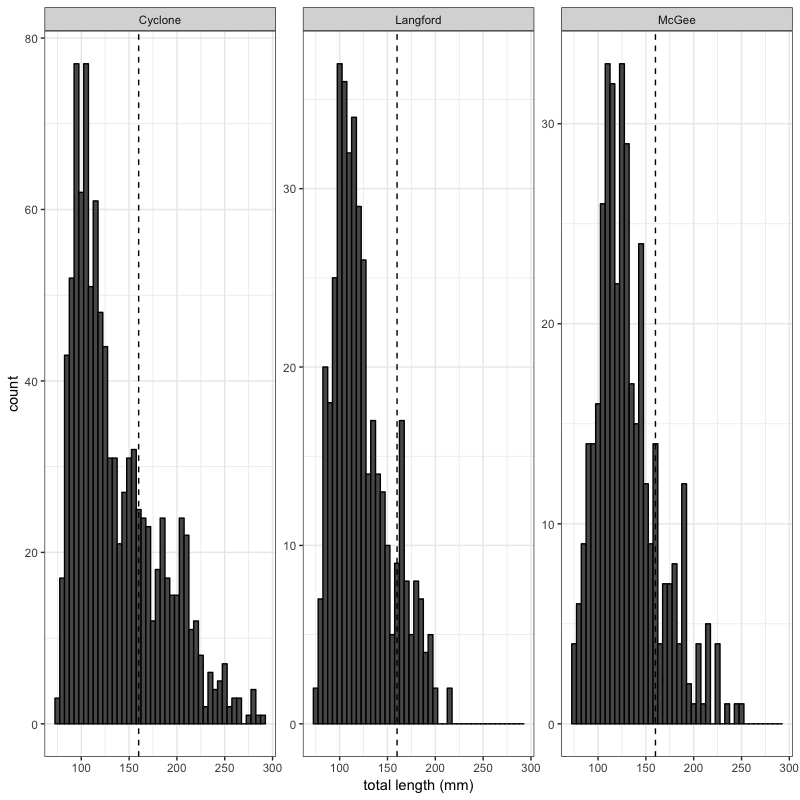


**Fig S4**: Length frequency distributions for each population for Cyclone, Langford, and McGee Creeks. The vertical dashed line at 160mm total length was the maximum starting length allowed for individuals in growth analyses. This was done to remove the influence of mature resident adults on growth rates.

**
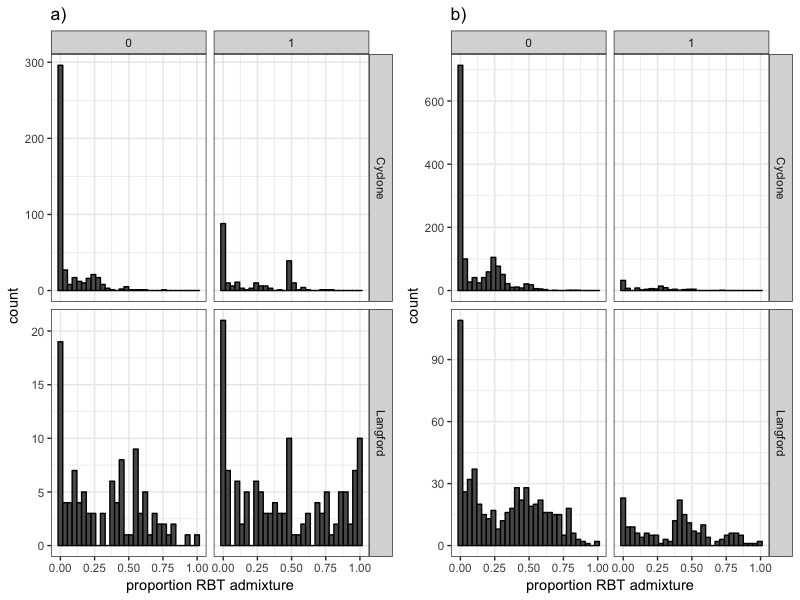
**

**Fig S5:** The distributions of proportion rainbow trout (RBT) admixture (pRBT) in adults (a) and juvenile datasets (b) sampled between 2013-2016 in Cyclone and Langford Creeks. Resident individuals (0) were sampled via electrofishing during July surveys and migratory individuals (1) were sampled via migrant fish traps from April-July.


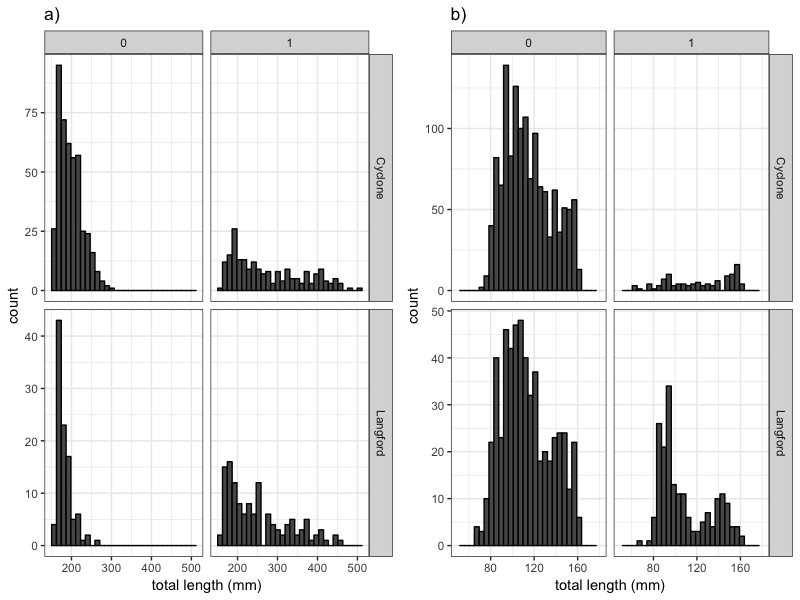


**Fig S6:** The length frequency distributions (TL, mm) of adults (a) and juveniles (b) sampled between 2013-2016 in Cyclone and Langford Creeks. Resident individuals (0) were sampled via electrofishing during July surveys and migratory individuals (1) were sampled via migrant fish traps from April-July.


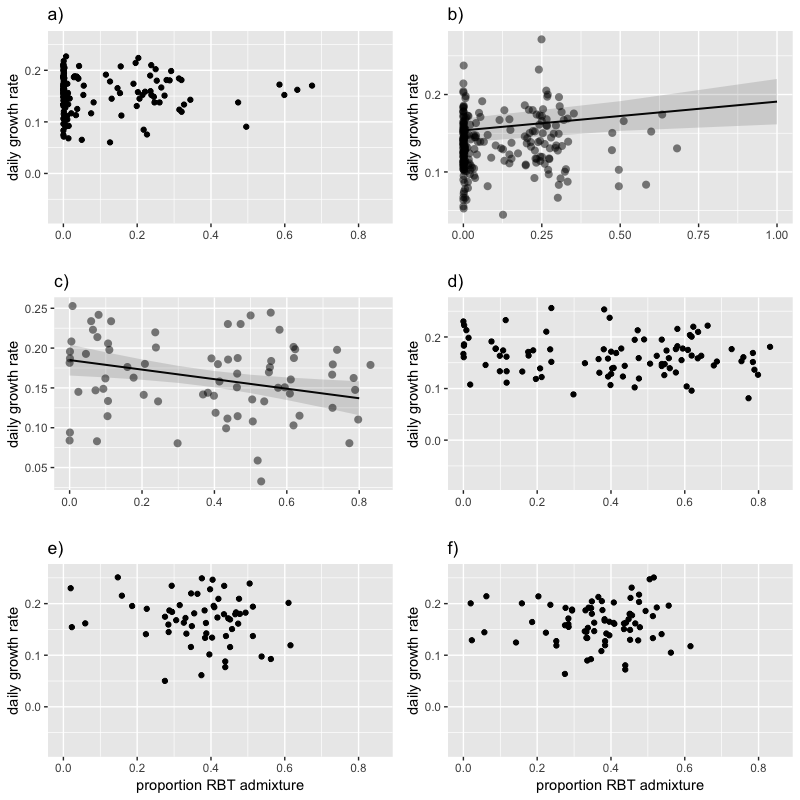


**Fig S7**: Relationship between daily growth rates (mm/d) and proportion rainbow trout (RBT) admixture (pRBT) throughout the spring (left column) and annually (right column). Cyclone Creek had no significant effect of pRBT on spring growth rates (a) and a significant positive effect of pRBT on annual growth rates (b). Langford Creek had a significant negative effect of pRBT on spring growth rates (c), but no significant effect of pRBT on annual growth rates (d). McGee Creek showed no significant effect of pRBT on spring or annual growth rates in length (e & f). However, McGee Creek did show a significant negative effect of PRBT on spring growth rates in mass (g/d; see Table S9).

**Table S1**: Summary statistics of environmental data collected at each study site from 2013-2017. We measured stream temperature using HOBO dataloggers and then estimated summer daily median temperature (ºC), spring growing-degree-days (GDD), and annual GDD for each population and year. We also measured stream flow (m^3^/s) through the year at each site and estimated summer base flow, mean spring flow, and maximum spring flow. Finally, we estimated *Oncorhynchus ssp.* density (# fish/m^2^) from multi-pass depletion estimates of abundance and stream width measurements from each population taken every July.

|  |  | **seasonal temperature metrics** | | | **flow metrics (m^3/sec)** | | |  |
| --- | --- | --- | --- | --- | --- | --- | --- | --- |
| **population** | **year** | **summer median** | **spring GDD** | **annual GDD** | **summer base** | **spring mean** | **spring max** | **summer density** |
| **Cyclone Creek** | **2013** | 13.61 | - | - | 0.179 | - | - | 0.12 |
|  | **2014** | 11.33 | 640 | 1250.5 | 0.437 | 1.884 | 2.837 | 0.16 |
|  | **2015** | 11.73 | 733.6 | 1367.2 | 0.0828 | 0.559 | 1.126 | 0.15 |
|  | **2016** | 10.4 | 771.5 | 1380.1 | 0.1023 | 0.552 | 1.124 | 0.22 |
|  | **2017** | - | 648 | 1210.8 | - | - | - | - |
| **Langford Creek** | **2013** | 10.34 | - | - | 0.157 | - | - | 0.16 |
|  | **2014** | 10.24 | 333.4 | 771.7 | 0.145 | 0.389 | 0.729 | 0.13 |
|  | **2015** | 10.66 | 317.5 | 897 | 0.081 | 0.136 | 0.223 | 0.08 |
|  | **2016** | 11.45 | 407.2 | 1034.4 | 0.0976 | 0.075 | 0.174 | 0.08 |
|  | **2017** |  | 397.3 | 1063.8 | - | - | - | - |
| **McGee Creek** | **2013** | - | - | - | - | - | - | - |
|  | **2014** | 12.11 | - | - | 0.123 | - | - | 0.05 |
|  | **2015** | 13.63 | 544.5 | 1385.2 | 0.0833 | 0.343 | 0.667 | 0.05 |
|  | **2016** | 14.29 | 504.9 | 1340.7 | 0.1628 | 0.902 | 3.714 | 0.03 |
|  | **2017** | - | 484.3 | 1300.5 | - | - | - | - |

**Table S2:** Correlation matrix of environmental covariates considered in the linear modelling of summer growth rates.

|  | stdmedianT | stdd7maxT | stdgdd | stdflow | stdflow.d | stddensity |
| --- | --- | --- | --- | --- | --- | --- |
| stdmedianT | 1 |  |  |  |  |  |
| stdd7maxT | 0.51 | 1 |  |  |  |  |
| stdgdd | **0.872** | 0.535 | 1 |  |  |  |
| stdflow | 0.051 | 0.102 | -0.052 | 1 |  |  |
| stdflow.d | -0.333 | 0.24 | -0.318 | -0.167 | 1 |  |
| stddensity | **-0.74** | -0.37 | **-0.74** | 0.034 | 0.388 | 1 |

**Table S3:** Correlation matrix of environmental covariates considered in the linear modelling of spring growth rates.

|  | std.total.gdd | std.meanT | std.max.meanT | std.med.flow | std.max.flow |
| --- | --- | --- | --- | --- | --- |
| std.total.gdd | 1 |  |  |  |  |
| std.meanT | **0.904** | 1 |  |  |  |
| std.max.meanT | **0.914** | **0.978** | 1 |  |  |
| std.med.flow | 0.397 | 0.517 | 0.46 | 1 |  |
| std.max.flow | 0.214 | 0.286 | 0.191 | **0.751** | 1 |

Table S4: Supported models considered in our AICc model selection for summer growth rates for length (a) and mass (b) in Cyclone Creek. We only considered models that contained only significant parameters (*p =* 0.05). The models below are ranked by AICc and the coefficients for these competing models are shown. Coefficients are shown for models that contain covariates for stream flow (flow), relative condition factor (K), median summer temperature (MedT), proportion rainbow trout (RBT) admixture (pRBT), total length (TL), weight (W), density (D), and any combination of interactions between these covariates that were supported. The coefficient for the intercepts is also shown (INT) and the number of parameters (PAR) for each model. The top model is bold and underlined.

**a)**

| **Cyclone Length** | **INT** | **flow** | **K** | **MedT** | **pRBT** | **TL** | **pRBT * MedT** | **pRBT * TL** | **D** | **PAR** | **logLik** | **AICc** | **delta** | **weight** |
| --- | --- | --- | --- | --- | --- | --- | --- | --- | --- | --- | --- | --- | --- | --- |
| **1** | **0.098** | **0.011** | **0.013** | **0.033** | **0.032** | **0.005** | **0.015** | **0.024** |  | **13** | **454.535** | **-881.691** | **0.000** | **0.776** |
| 2 | 0.092 | 0.011 | 0.013 | 0.023 | 0.024 | 0.004 |  | 0.022 |  | 12 | 452.081 | -878.984 | 2.707 | 0.201 |
| 3 | 0.086 | 0.010 | 0.013 | 0.023 | 0.014 | -0.012 |  |  |  | 11 | 448.589 | -874.186 | 7.505 | 0.018 |
| 4 | 0.090 | 0.011 | 0.015 | 0.023 | 0.012 |  |  |  |  | 10 | 445.965 | -871.105 | 10.586 | 0.004 |
| 5 | 0.082 | 0.011 | 0.017 | 0.022 |  |  |  |  |  | 9 | 442.822 | -866.972 | 14.720 | 0.000 |
| 10 | 0.102 |  | 0.014 |  | 0.023 | 0.002 |  | 0.021 | -0.025 | 11 | 444.867 | -866.741 | 14.951 | 0.000 |
| 11 | 0.097 |  | 0.014 |  | 0.014 | -0.013 |  |  | -0.026 | 10 | 441.796 | -862.768 | 18.923 | 0.000 |
| 12 | 0.101 |  | 0.016 |  | 0.011 |  |  |  | -0.025 | 9 | 438.510 | -858.348 | 23.343 | 0.000 |
| 6 | 0.080 |  | 0.018 | 0.023 |  |  |  |  |  | 8 | 436.862 | -857.189 | 24.503 | 0.000 |
| 9 | 0.083 | 0.010 | 0.017 | 0.023 |  |  |  |  |  | 5 | 433.593 | -856.966 | 24.725 | 0.000 |
| 13 | 0.092 |  | 0.019 |  |  |  |  |  | -0.023 | 8 | 435.838 | -855.141 | 26.551 | 0.000 |
| 7 | 0.070 |  | 0.021 |  |  |  |  |  |  | 7 | 425.908 | -837.400 | 44.291 | 0.000 |
| 14 | 0.070 |  | 0.021 |  |  |  |  |  |  | 7 | 425.908 | -837.400 | 44.291 | 0.000 |
| 8 | 0.068 |  |  |  |  |  |  |  |  | 6 | 407.171 | -802.031 | 79.660 | 0.000 |

**b)**

| **Cyclone Mass** | **INT** | **K** | **pRBT** | **W** | **pRBT * W** | **MedT** | **D** | **PAR** | **logLik** | **AICc** | **delta** | **weight** |
| --- | --- | --- | --- | --- | --- | --- | --- | --- | --- | --- | --- | --- |
| **1** | **0.031** | **-0.006** | **0.037** | **0.034** | **0.055** |  |  | **10** | **609.154** | **-1197.483** | **0.000** | **0.963** |
| 2 | 0.031 |  | 0.034 | 0.035 | 0.054 |  |  | 9 | 604.818 | -1190.965 | 6.519 | 0.037 |
| 4 | 0.017 |  | 0.011 |  |  |  |  | 7 | 586.006 | -1157.598 | 39.886 | 0.000 |
| 3 | 0.015 |  | 0.011 | -0.003 |  |  |  | 8 | 586.190 | -1155.844 | 41.639 | 0.000 |
| 5 | 0.009 |  |  |  |  |  |  | 6 | 578.968 | -1145.627 | 51.857 | 0.000 |
| 6 | 0.005 |  |  |  |  | -0.011 |  | 3 | 559.340 | -1112.592 | 84.891 | 0.000 |
| 7 | 0.002 |  |  |  |  |  | 0.007 | 3 | 553.249 | -1100.410 | 97.074 | 0.000 |

Table S5: Supported models considered in our AICc model selection for summer growth rates for length (a) and mass (b) in Langford Creek. We only considered models that contained only significant parameters (*p =* 0.05). The models below are ranked by AICc and the coefficients for these competing models are shown. Coefficients are shown for models that contain covariates for stream flow (flow), relative condition factor (K), median summer temperature (MedT), proportion rainbow trout (RBT) admixture (pRBT), total length (TL), weight (W), density (D), and any combination of interactions between these covariates that were supported. The coefficient for the intercepts is also shown (INT) and the number of parameters (PAR) for each model. The top model is bold and underlined.

**a)**

| **Langford Length** | **INT** | **MedT** | **pRBT** | **pRBT * MedT** | **PAR** | **logLik** | **AICc** | **delta** | **weight** |
| --- | --- | --- | --- | --- | --- | --- | --- | --- | --- |
| **1** | **0.186** | **0.011** | **0.048** | **0.048** | **9** | **163.632** | **-307.900** | **0.000** | **0.583** |
| 4 | 0.173 |  |  |  | 6 | 159.448 | -306.273 | 1.626 | 0.258 |
| 3 | 0.187 | 0.015 |  |  | 7 | 159.763 | -304.689 | 3.210 | 0.117 |
| 2 | 0.188 | 0.019 | 0.003 |  | 8 | 159.865 | -302.647 | 5.252 | 0.042 |

**b)**

| **Langford Mass** | **INT** | **flow** | **K** | **MedT** | **pRBT** | **W** | **pRBT * flow** | **pRBT * MedT** | **D** | **PAR** | **logLik** | **AICc** | **delta** | **weight** |
| --- | --- | --- | --- | --- | --- | --- | --- | --- | --- | --- | --- | --- | --- | --- |
| **1** | **0.079** | **-0.039** | **-0.007** | **-0.001** | **0.048** | **0.041** | **0.022** | **0.053** |  | **13** | **232.637** | **-436.431** | **0.000** | **0.398** |
| 12 | 0.074 |  | -0.007 |  |  | 0.050 |  |  | -0.027 | 9 | 227.420 | -435.476 | 0.955 | 0.247 |
| 2 | 0.073 | -0.043 |  | -0.008 | 0.048 | 0.038 | 0.025 | 0.054 |  | 12 | 230.663 | -434.907 | 1.524 | 0.186 |
| 13 | 0.074 |  |  |  |  | 0.050 |  |  | -0.025 | 8 | 225.177 | -433.272 | 3.159 | 0.082 |
| 3 | 0.092 | -0.017 |  | 0.009 | 0.027 | 0.054 |  | 0.029 |  | 11 | 227.984 | -431.937 | 4.493 | 0.042 |
| 4 | 0.105 |  |  | 0.023 | 0.035 | 0.056 |  | 0.036 |  | 10 | 226.803 | -431.927 | 4.503 | 0.042 |
| 6 | 0.105 |  |  | 0.027 |  | 0.052 |  |  |  | 8 | 221.714 | -426.345 | 10.086 | 0.003 |
| 5 | 0.107 |  |  | 0.030 | 0.003 | 0.055 |  |  |  | 9 | 222.036 | -424.708 | 11.723 | 0.001 |
| 7 | 0.084 |  |  |  |  | 0.053 |  |  |  | 7 | 219.405 | -423.973 | 12.457 | 0.001 |
| 9 | 0.086 | -0.036 |  |  |  | 0.054 |  |  |  | 4 | 210.798 | -413.304 | 23.127 | 0.000 |
| 8 | 0.058 |  |  |  |  |  |  |  |  | 6 | 211.550 | -410.478 | 25.952 | 0.000 |
| 10 | 0.093 |  |  |  |  | 0.064 |  |  |  | 3 | 204.992 | -403.809 | 32.621 | 0.000 |
| 11 | 0.063 | -0.044 |  |  |  |  |  |  |  | 3 | 203.921 | -401.669 | 34.762 | 0.000 |

Table S6: Supported models considered in our AICc model selection for summer growth rates for length (a) and mass (b) in McGee Creek. We only considered models that contained only significant parameters (*p =* 0.05). The models below are ranked by AICc and the coefficients for these competing models are shown. Coefficients are shown for models that contain covariates for stream flow (flow), relative condition factor (K), median summer temperature (MedT), proportion rainbow trout (RBT) admixture (pRBT), total length (TL), weight (W), density (D), and any combination of interactions between these covariates that were supported. The coefficient for the intercepts is also shown (INT) and the number of parameters (PAR) for each model. The top model is bold and underlined.

**a)**

| **McGee Length** | **INT** | **pRBT** | **year** | **PAR** | **logLik** | **AICc** | **delta** | **weight** |
| --- | --- | --- | --- | --- | --- | --- | --- | --- |
| **1** | **0.132** | **0.019** |  | **6** | **192.556** | **-372.507** | **0.000** | **0.948** |
| 2 | 0.142 |  |  | 5 | 188.424 | -366.420 | 6.087 | 0.045 |
| 3 | 0.157 |  | + | 4 | 185.494 | -362.704 | 9.803 | 0.007 |

**b)**

| **McGee Mass** | **INT** | **MedT** | **pRBT** | **flow** | **D** | **PAR** | **logLik** | **AICc** | **delta** | **weight** |
| --- | --- | --- | --- | --- | --- | --- | --- | --- | --- | --- |
| 6 | -0.078 |  | 0.011 |  | -0.087 | 7 | 257.303 | -499.795 | 0.000 | 0.761 |
| **1** | **0.005** | **0.019** | **0.011** |  |  | **7** | **255.709** | **-496.607** | **3.188** | **0.155** |
| 7 | -0.067 |  |  |  | -0.081 | 6 | 253.778 | -494.951 | 4.843 | 0.068 |
| 2 | 0.030 |  | 0.010 |  |  | 6 | 251.513 | -490.422 | 9.373 | 0.007 |
| 4 | 0.015 | 0.018 |  | 0.012 |  | 4 | 248.942 | -489.600 | 10.195 | 0.005 |
| 5 | 0.010 | 0.021 |  |  |  | 3 | 247.619 | -489.070 | 10.725 | 0.004 |
| 3 | 0.034 |  |  |  |  | 5 | 249.072 | -487.715 | 12.080 | 0.002 |

Table S7: Supported models considered in our AICc model selection for spring growth rates for length (a) and mass (b) in Cyclone Creek. We only considered models that contained only significant parameters (*p =* 0.05). The models below are ranked by AICc and the coefficients for these competing models are shown. Coefficients are shown for models that contain covariates for relative condition factor (K), spring growing-degree-days (GDD), proportion rainbow trout (RBT) admixture (pRBT), total length (TL), weight (W), and any combination of interactions between these covariates that were supported. The coefficient for the intercepts is also shown (INT) and the number of parameters (PAR) for each model. The top model is bold and underlined.

**a)**

| **Cyclone Length** | **INT** | **K** | **GDD** | **TL** | **PAR** | **logLik** | **AICc** | **delta** | **weight** |
| --- | --- | --- | --- | --- | --- | --- | --- | --- | --- |
| **1** | **0.131** | **-0.005** | **0.016** | **-0.023** | **5** | **289.766** | **-569.101** | **0.000** | **0.667** |
| 2 | 0.145 | -0.004 |  | -0.022 | 4 | 287.522 | -566.758 | 2.343 | 0.207 |
| 3 | 0.145 |  |  | -0.022 | 3 | 285.976 | -565.781 | 3.320 | 0.127 |
| 4 | 0.155 |  |  |  | 2 | 276.292 | -548.500 | 20.600 | 0.000 |

**b)**

| **Cyclone Mass** | **INT** | **K** | **W** | **PAR** | **logLik** | **AICc** | **delta** | **weight** |
| --- | --- | --- | --- | --- | --- | --- | --- | --- |
| **1** | **0.105** | **-0.009** | **0.052** | **4** | **317.573** | **-626.861** | **0.000** | **0.999** |
| 2 | 0.103 |  | 0.045 | 3 | 308.461 | -610.752 | 16.109 | 0.000 |
| 4 | 0.085 | -0.006 |  | 3 | 296.417 | -586.664 | 40.197 | 0.000 |
| 3 | 0.085 |  |  | 2 | 293.107 | -582.130 | 44.731 | 0.000 |

Table S8: Supported models considered in our AICc model selection for spring growth rates for length (a) and mass (b) in Langford Creek. We only considered models that contained only significant parameters (*p =* 0.05). The models below are ranked by AICc and the coefficients for these competing models are shown. Coefficients are shown for models that contain covariates for relative condition factor (K), spring growing-degree-days (GDD), proportion rainbow trout (RBT) admixture (pRBT), total length (TL), weight (W), and any combination of interactions between these covariates that were supported. The coefficient for the intercepts is also shown (INT) and the number of parameters (PAR) for each model. The top model is bold and underlined.

**a)**

| **Langford Length** | **INT** | **pRBT** | **GDD** | **PAR** | **logLik** | **AICc** | **delta** | **weight** |
| --- | --- | --- | --- | --- | --- | --- | --- | --- |
| **1** | **0.263** | **-0.013** | **0.065** | **4** | **127.627** | **-246.683** | **0.000** | **0.822** |
| 2 | 0.222 |  | 0.042 | 3 | 124.102 | -241.866 | 4.817 | 0.074 |
| 3 | 0.162 |  |  | 2 | 122.119 | -240.071 | 6.612 | 0.030 |

**b)**

| **Langford Mass** | **INT** | **K** | **pRBT** | **GDD** | **W** | **PAR** | **logLik** | **AICc** | **delta** | **weight** |
| --- | --- | --- | --- | --- | --- | --- | --- | --- | --- | --- |
| **1** | **0.268** | **-0.016** | **-0.011** | **0.085** | **0.090** | **6** | **140.225** | **-267.216** | **0.000** | **0.890** |
| 2 | 0.239 | -0.018 |  | 0.069 | 0.094 | 5 | 136.927 | -262.984 | 4.232 | 0.107 |
| 3 | 0.141 | -0.010 |  |  | 0.090 | 4 | 131.306 | -254.040 | 13.176 | 0.001 |
| 6 | 0.141 | -0.010 |  |  | 0.090 | 4 | 131.306 | -254.040 | 13.176 | 0.001 |
| 4 | 0.136 |  |  |  | 0.078 | 3 | 129.115 | -251.892 | 15.324 | 0.000 |
| 7 | 0.136 |  |  |  | 0.078 | 3 | 129.115 | -251.892 | 15.324 | 0.000 |
| 8 | 0.175 |  |  | 0.047 |  | 3 | 121.504 | -236.671 | 30.545 | 0.000 |
| 5 | 0.110 |  |  |  |  | 2 | 119.266 | -234.365 | 32.851 | 0.000 |

Table S9: Supported models considered in our AICc model selection for spring growth rates for length (a) and mass (b) in McGee Creek. We only considered models that contained only significant parameters (*p =* 0.05). The models below are ranked by AICc and the coefficients for these competing models are shown. Coefficients are shown for models that contain covariates for relative condition factor (K), spring growing-degree-days (GDD), proportion rainbow trout (RBT) admixture (pRBT), total length (TL), weight (W), and any combination of interactions between these covariates that were supported. The coefficient for the intercepts is also shown (INT) and the number of parameters (PAR) for each model. The top model is bold and underlined.

**a)**

| **McGee Length** | **INT** | **TL** | **PAR** | **logLik** | **AICc** | **delta** | **weight** |
| --- | --- | --- | --- | --- | --- | --- | --- |
| **1** | **0.161** | **-0.021** | **5** | **108.544** | **-205.998** | **0.000** | **0.923** |
| 2 | 0.167 |  | 4 | 104.877 | -201.040 | 4.958 | 0.077 |

**b)**

| **McGee Mass** | **INT** | **pRBT** | **W** | **K** | **PAR** | **logLik** | **AICc** | **delta** | **weight** |
| --- | --- | --- | --- | --- | --- | --- | --- | --- | --- |
| **1** | **0.146** | **-0.019** | **0.061** |  | **4** | **113.244** | **-217.775** | **0.000** | **0.520** |
| 4 | 0.132 |  | 0.063 | -0.015 | 6 | 115.202 | -216.849 | 0.925 | 0.328 |
| 2 | 0.132 |  | 0.060 |  | 3 | 110.864 | -215.308 | 2.467 | 0.152 |
| 3 | 0.116 |  |  |  | 2 | 104.157 | -204.106 | 13.668 | 0.001 |

Table S10: Supported models considered in our AICc model selection for annual growth rates for length (a) and mass (b) in Cyclone Creek. We only considered models that contained only significant parameters (*p =* 0.05). The models below are ranked by AICc and the coefficients for these competing models are shown. Coefficients are shown for models that contain covariates for year, relative condition factor (K), annual growing-degree-days (GDD), proportion rainbow trout (RBT) admixture (pRBT), total length (TL), weight (W), and any combination of interactions between these covariates that were supported. The coefficient for the intercepts is also shown (INT) and the number of parameters (PAR) for each model. The top model is bold and underlined.

**a)**

| **Cyclone Length** | **INT** | **K** | **pRBT** | **TL** | **year** | **pRBT * K** | **PAR** | **logLik** | **AICc** | **delta** | **weight** |
| --- | --- | --- | --- | --- | --- | --- | --- | --- | --- | --- | --- |
| **1** | **0.149** | **-0.007** | **0.008** | **-0.024** | **+** | **-0.012** | **9** | **513.648** | **-1008.516** | **0.000** | **1.000** |
| 7 | 0.146 |  |  | -0.023 | + |  | 6 | 501.167 | -989.975 | 18.541 | 0.000 |
| 2 | 0.129 | -0.009 | 0.008 | -0.025 |  | -0.014 | 6 | 500.201 | -988.042 | 20.474 | 0.000 |
| 4 | 0.127 |  | 0.005 | -0.024 |  |  | 4 | 486.561 | -964.953 | 43.563 | 0.000 |
| 3 | 0.127 | -0.002 | 0.006 | -0.025 |  |  | 5 | 487.326 | -964.396 | 44.120 | 0.000 |
| 5 | 0.125 |  |  | -0.024 |  |  | 3 | 484.969 | -963.837 | 44.679 | 0.000 |
| 6 | 0.137 |  |  |  |  |  | 2 | 471.112 | -938.174 | 70.342 | 0.000 |

**b)**

| **Cyclone Mass** | **INT** | **GDD** | **pRBT** | **W** | **year** | **pRBT * GDD** | **PAR** | **logLik** | **AICc** | **delta** | **weight** |
| --- | --- | --- | --- | --- | --- | --- | --- | --- | --- | --- | --- |
| **1** | **0.123** | **-0.024** | **0.001** | **0.063** | **+** | **0.022** | **9** | **535.797** | **-1052.814** | **0.000** | **0.958** |
| 2 | 0.099 | 0.023 | 0.000 | 0.061 |  | 0.024 | 6 | 529.316 | -1046.273 | 6.541 | 0.036 |
| 7 | 0.123 | -0.039 |  | 0.065 | + |  | 7 | 528.024 | -1041.567 | 11.247 | 0.003 |
| 8 | 0.118 |  |  | 0.065 | + |  | 6 | 525.929 | -1039.498 | 13.316 | 0.001 |
| 3 | 0.103 | 0.012 | 0.006 | 0.061 |  |  | 5 | 523.740 | -1037.226 | 15.589 | 0.000 |
| 4 | 0.101 | 0.013 |  | 0.063 |  |  | 4 | 520.786 | -1033.403 | 19.411 | 0.000 |
| 5 | 0.105 |  |  | 0.064 |  |  | 3 | 517.381 | -1028.660 | 24.154 | 0.000 |
| 6 | 0.075 |  |  |  |  |  | 2 | 487.638 | -971.225 | 81.590 | 0.000 |

Table S11: Supported models considered in our AICc model selection for annual growth rates for length (a) and mass (b) in Langford Creek. We only considered models that contained only significant parameters (*p =* 0.05). The models below are ranked by AICc and the coefficients for these competing models are shown. Coefficients are shown for models that contain covariates for year, relative condition factor (K), annual growing-degree-days (GDD), proportion rainbow trout (RBT) admixture (pRBT), total length (TL), weight (W), and any combination of interactions between these covariates that were supported. The coefficient for the intercepts is also shown (INT) and the number of parameters (PAR) for each model. The top model is bold and underlined.

**a)**

| **Langford Length** | **INT** | **GDD** | **K** | **TL** | **year** | **PAR** | **logLik** | **AICc** | **delta** | **weight** |
| --- | --- | --- | --- | --- | --- | --- | --- | --- | --- | --- |
| **1** | **0.000** | **-0.063** | **-0.012** | **-0.030** | **+** | **8** | **186.084** | **-354.473** | **0.000** | **0.578** |
| 4 | 0.149 |  |  | -0.027 |  | 3 | 179.224 | -352.181 | 2.293 | 0.184 |
| 3 | 0.160 | 0.009 |  | -0.028 |  | 4 | 179.937 | -351.425 | 3.048 | 0.126 |
| 2 | 0.060 | -0.038 |  | -0.027 | + | 7 | 183.202 | -351.102 | 3.371 | 0.107 |
| 5 | 0.164 |  |  |  |  | 2 | 174.747 | -345.362 | 9.111 | 0.006 |

**b)**

| **Langford Mass** | **INT** | **GDD** | **K** | **W** | **year** | **PAR** | **logLik** | **AICc** | **delta** | **weight** |
| --- | --- | --- | --- | --- | --- | --- | --- | --- | --- | --- |
| **1** | **-0.016** | **-0.070** | **-0.019** | **0.087** | **+** | **11** | **194.143** | **-363.067** | **0.000** | **0.931** |
| 2 | 0.128 |  | -0.014 | 0.086 | + | 10 | 190.228 | -357.806 | 5.261 | 0.067 |
| 3 | 0.149 |  | -0.006 | 0.094 |  | 7 | 182.457 | -349.612 | 13.455 | 0.001 |
| 4 | 0.150 |  |  | 0.093 |  | 6 | 181.196 | -349.426 | 13.641 | 0.001 |
| 5 | 0.102 |  |  |  |  | 5 | 171.328 | -331.974 | 31.093 | 0.000 |

Table S12: Supported models considered in our AICc model selection for annual growth rates for length (a) and mass (b) in McGee Creek. We only considered models that contained only significant parameters (*p =* 0.05). The models below are ranked by AICc and the coefficients for these competing models are shown. Coefficients are shown for models that contain covariates for year, relative condition factor (K), annual growing-degree-days (GDD), proportion rainbow trout (RBT) admixture (pRBT), total length (TL), weight (W), and any combination of interactions between these covariates that were supported. The coefficient for the intercepts is also shown (INT) and the number of parameters (PAR) for each model. The top model is bold and underlined.

**a)**

| **McGeeLength** | **INT** | **TL** | **year** | **PAR** | **logLik** | **AICc** | **delta** | **weight** |
| --- | --- | --- | --- | --- | --- | --- | --- | --- |
| **1** | **0.153** | **-0.024** |  | **3** | **138.919** | **-271.509** | **0.000** | **0.755** |
| 3 | 0.157 |  | + | 4 | 138.349 | -268.143 | 3.366 | 0.140 |
| 2 | 0.164 |  |  | 2 | 135.856 | -267.550 | 3.959 | 0.104 |

**b)**

| **McGee Mass** | **INT** | **W** | **PAR** | **logLik** | **AICc** | **delta** | **weight** |
| --- | --- | --- | --- | --- | --- | --- | --- |
| **1** | **0.153** | **0.107** | **3** | **147.371** | **-288.413** | **0.000** | **1.000** |
| 2 | 0.105 |  | 2 | 134.069 | -263.975 | 24.438 | 0.000 |

Table S13: Supported models considered in our AICc model selection for the GLM analysis of migratory life history strategy of juvenile *Oncorhynchus spp.* in Cyclone and Langford Creeks. We only considered models that contained only significant parameters (*p =* 0.05). The models below are ranked by AICc and the coefficients for these competing models are shown. Coefficients are shown for models that contain covariates for year, relative condition factor (K), annual growing-degree-days (GDD), proportion rainbow trout (RBT) admixture (pRBT), total length (TL), weight (W), and any combination of interactions between these covariates that were supported. The coefficient for the intercepts is also shown (INT) and the number of parameters (PAR) for each model. The top model is bold and underlined.

| **Cyclone Juveniles** | **INT** | **K** | **pRBT** | **TL** | **year** | **pRBT * TL** | **PAR** | **logLik** | **AICc** | **delta** | **weight** |
| --- | --- | --- | --- | --- | --- | --- | --- | --- | --- | --- | --- |
| **1** | **-1.688** | **-0.912** | **0.279** | **1.118** | **+** | **-0.737** | **8** | **-340.110** | **696.321** | **0.000** | **0.964** |
| 2 | -1.615 | -0.901 | 0.624 | 1.018 | + |  | 7 | -344.434 | 702.945 | 6.624 | 0.035 |
| 3 | -2.015 | -0.939 | 0.620 | 0.975 |  |  | 4 | -351.577 | 711.181 | 14.861 | 0.001 |
| 4 | -2.428 | -1.006 | 0.590 |  |  |  | 3 | -357.962 | 721.941 | 25.620 | 0.000 |
| 6 | -1.863 | -0.474 |  | 0.883 | + |  | 6 | -363.580 | 739.218 | 42.897 | 0.000 |
| 5 | -2.612 |  | 0.441 |  |  |  | 2 | -370.606 | 745.220 | 48.899 | 0.000 |
| 10 | -2.046 | -0.562 |  | 0.812 |  |  | 3 | -370.978 | 747.972 | 51.651 | 0.000 |
| 8 | -2.106 |  |  | 0.943 |  |  | 2 | -375.229 | 754.467 | 58.146 | 0.000 |
| 7 | -2.557 |  |  |  | + |  | 4 | -373.775 | 755.577 | 59.257 | 0.000 |
| 9 | -2.394 | -0.646 |  |  |  |  | 2 | -375.930 | 755.868 | 59.548 | 0.000 |
| **Langford Juveniles** | **INT** | **K** | **pRBT** | **TL** | **year** | **pRBT * TL** | **PAR** | **logLik** | **AICc** | **delta** | **weight** |
| **1** | **-1.437** | **-0.732** | **-0.247** | **-0.820** | **+** | **-1.771** | **8** | **-306.403** | **629.000** | **0.000** | **1.000** |
| 3 | -0.921 | -0.752 | 0.530 |  | + |  | 6 | -331.278 | 674.669 | 45.669 | 0.000 |
| 2 | -1.056 | -0.739 | 0.505 | -0.274 | + |  | 7 | -330.591 | 675.333 | 46.333 | 0.000 |
| 6 | -1.282 | -0.694 |  | -0.497 | + |  | 6 | -342.112 | 696.336 | 67.336 | 0.000 |
| 4 | -1.039 | -0.711 |  |  | + |  | 5 | -344.599 | 699.278 | 70.278 | 0.000 |
| 5 | -0.916 |  |  |  | + |  | 4 | -372.652 | 753.357 | 124.357 | 0.000 |
| 9 | -1.137 | -0.769 |  | -0.334 |  |  | 3 | -388.899 | 783.829 | 154.829 | 0.000 |
| 8 | -0.989 | -0.782 |  |  |  |  | 2 | -390.221 | 784.457 | 155.457 | 0.000 |
| 7 | -1.284 |  |  | -0.471 |  |  | 2 | -424.629 | 853.274 | 224.274 | 0.000 |

Table S14: Supported models considered in our AICc model selection for the GLM analysis of migratory life history strategy of adult *Oncorhynchus spp.* in Cyclone and Langford Creeks. We only considered models that contained only significant parameters (*p =* 0.05). The models below are ranked by AICc and the coefficients for these competing models are shown. Coefficients are shown for models that contain covariates for year, relative condition factor (K), annual growing-degree-days (GDD), proportion rainbow trout (RBT) admixture (pRBT), total length (TL), weight (W), and any combination of interactions between these covariates that were supported. The coefficient for the intercepts is also shown (INT) and the number of parameters (PAR) for each model. The top model is bold and underlined.

| **Cyclone Adults** | **INT** | **K** | **pRBT** | **TL** | **year** | **PAR** | **logLik** | **AICc** | **delta** | **weight** |
| --- | --- | --- | --- | --- | --- | --- | --- | --- | --- | --- |
| **1** | **-1.446** | **-2.078** | **0.545** | **1.482** | **+** | **7** | **-234.219** | **482.611** | **0.000** | **1.000** |
| 2 | -1.527 | -1.872 |  | 1.598 | + | 6 | -243.773 | 499.675 | 17.064 | 0.000 |
| 6 | -2.698 | -1.661 |  | 1.635 |  | 3 | -260.450 | 526.938 | 44.326 | 0.000 |
| 4 | -2.784 |  |  | 1.504 |  | 2 | -298.566 | 601.150 | 118.539 | 0.000 |
| 5 | -0.568 | -1.226 |  |  |  | 2 | -377.384 | 758.785 | 276.174 | 0.000 |
| 3 | 0.148 |  |  |  | + | 4 | -392.434 | 792.929 | 310.318 | 0.000 |
| **Langford Adults** | **INT** | **K** | **pRBT** | **TL** | **year** | **PAR** | **logLik** | **AICc** | **delta** | **weight** |
| **1** | **-0.485** | **-2.075** | **0.597** | **1.968** | **+** | **7** | **-75.103** | **164.708** | **0.000** | **0.968** |
| 3 | -0.135 | -1.971 |  | 1.751 | + | 6 | -80.077 | 172.528 | 7.820 | 0.019 |
| 2 | -1.818 | -1.873 | 0.601 | 1.905 |  | 4 | -82.619 | 173.414 | 8.706 | 0.012 |
| 7 | -1.514 | -1.785 |  | 1.696 |  | 3 | -88.500 | 183.107 | 18.399 | 0.000 |
| 6 | 0.724 | -2.157 |  |  |  | 2 | -113.537 | 231.126 | 66.418 | 0.000 |
| 5 | -2.223 |  |  | 1.928 |  | 2 | -114.145 | 232.342 | 67.634 | 0.000 |
| 4 | 1.649 |  |  |  | + | 4 | -148.480 | 305.136 | 140.428 | 0.000 |
